# Single shot acquisition of spatially resolved spin wave dispersion relations using X-ray microscopy

**DOI:** 10.1038/s41598-020-74785-4

**Published:** 2020-10-23

**Authors:** Nick Träger, Felix Groß, Johannes Förster, Korbinian Baumgaertl, Hermann Stoll, Markus Weigand, Gisela Schütz, Dirk Grundler, Joachim Gräfe

**Affiliations:** 1 Max Planck Institute for Intelligent Systems, 70569 Stuttgart, Germany; 2 Laboratory of Nanoscale Magnetic Materials and Magnonics, Institute of Materials, EPFL, 1015 Lausanne, Switzerland; 3 Institute of Physics, Johannes Gutenberg-University Mainz, 55099 Mainz, Germany; 4 Helmholtz-Zentrum Berlin für Materialien und Energie GmbH, 12489 Berlin, Germany

**Keywords:** Imaging techniques, Spintronics, Magnetic properties and materials, Physics, Condensed-matter physics, Ferromagnetism

## Abstract

For understanding magnonic materials the fundamental characterization of their frequency response is essential. However, determining full dispersion relations and real space wavelength measurements are challenging and time-consuming tasks. We present an approach for spin wave excitation by a modified Sinc pulse, which combines a cosine signal with a conventional Sinc function. The resulting adjustable frequency bands lead to a broadband spin wave excitation at uniform power levels. Subsequently, time resolved scanning transmission X-ray microscopy is used for direct imaging of all excited spin waves in real space. To demonstrate the capabilities of this approach, a modified Sinc excitation of an ultra-thin yttrium-iron-garnet film is shown that simultaneously reveals phase, amplitude, and *k*-space information from a single measurement. Consequently, this approach allows a fast and thorough access to the full dispersion relation including spatial maps of the individual spin wave modes, enabling complete characterization of magnonic materials down to the nanoscale in real and reciprocal space.

Magnonics, which describes the collective precessional motion of local magnetic moments, is one of the most intriguing phenomena in the world of nano magnetism^1–5^. Over the last two decades spin wave generation in various materials and geometries has been intensively studied revealing, for example, spin wave wavelengths in the nanometer regime, magnonic waveguides, and filters or spin wave interference effects realizing magnonic logic devices^6–10^. Experiments and theory have shown that magnonic structures exhibit highly anisotropic dispersion relations^1,5^. Therefore, knowing the exact dispersion is a crucial factor in designing and characterizing nanoscaled devices and their prospective capabilities in magnonics applications.

Common experimental methods to obtain dispersion relations *f*(*k*), i.e. frequency *f* versus wavevector *k*, are *k*-sensitive Brillouin light scattering (BLS) or time resolved magneto-optical Kerr effect (TR-MOKE) measurements^11,12^. However, their spatial resolution is limited by the used probing wavelengths. Thus, spin wave detection with *k*-sensitivity below wavelengths of $\lambda < 250\hbox { nm}$ is not possible. We address the challenge of determining the full dispersion relation for a large range of frequencies and wavevectors combined with real space imaging. This is achieved by transitioning to time resolved scanning transmission X-ray microscopy (STXM) with high spatial ($<20\hbox { nm}$) and temporal ($<35\hbox { ps}$) resolution , which can detect propagating magnons in the sub-$100\hbox { nm}$ regime with both phase and amplitude information simultaneously^6,7,13–17^.

**Figure 1:**
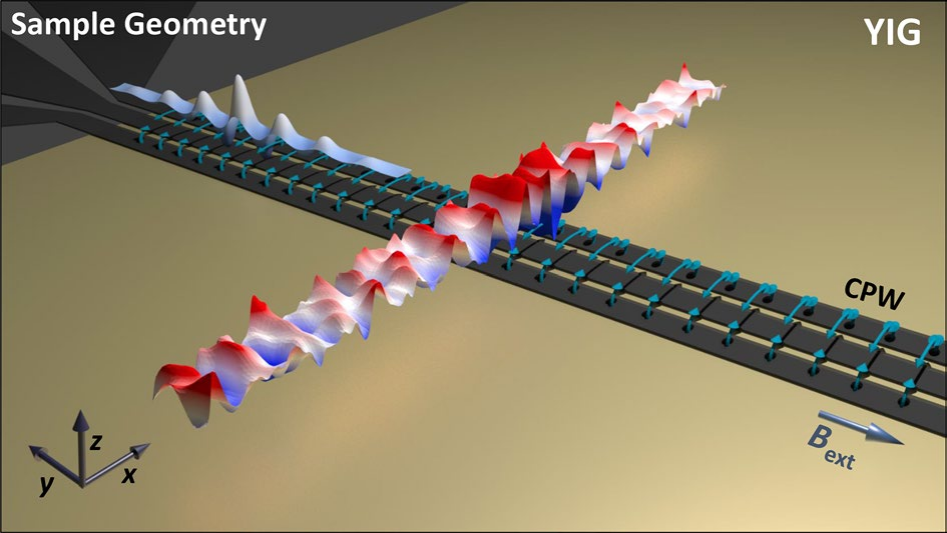
Schematic illustration of the sample geometry. A CPW (grey) on top of an ultra-thin YIG film induces spin waves by RF currents. The blue arrows represents the applied oscillating field. The modified Sinc excitation is indicated on the left side. An external field $B_{\mathrm {ext}}$ can be applied parallel to the CPW. Spin waves propagate perpendicular to $B_{\mathrm {ext}}$ along the *x*-direction in case of Damon–Eshbach modes. The CPW transfers wavevectors $k_{x}$ in *x*-directions.

**Figure 2:**
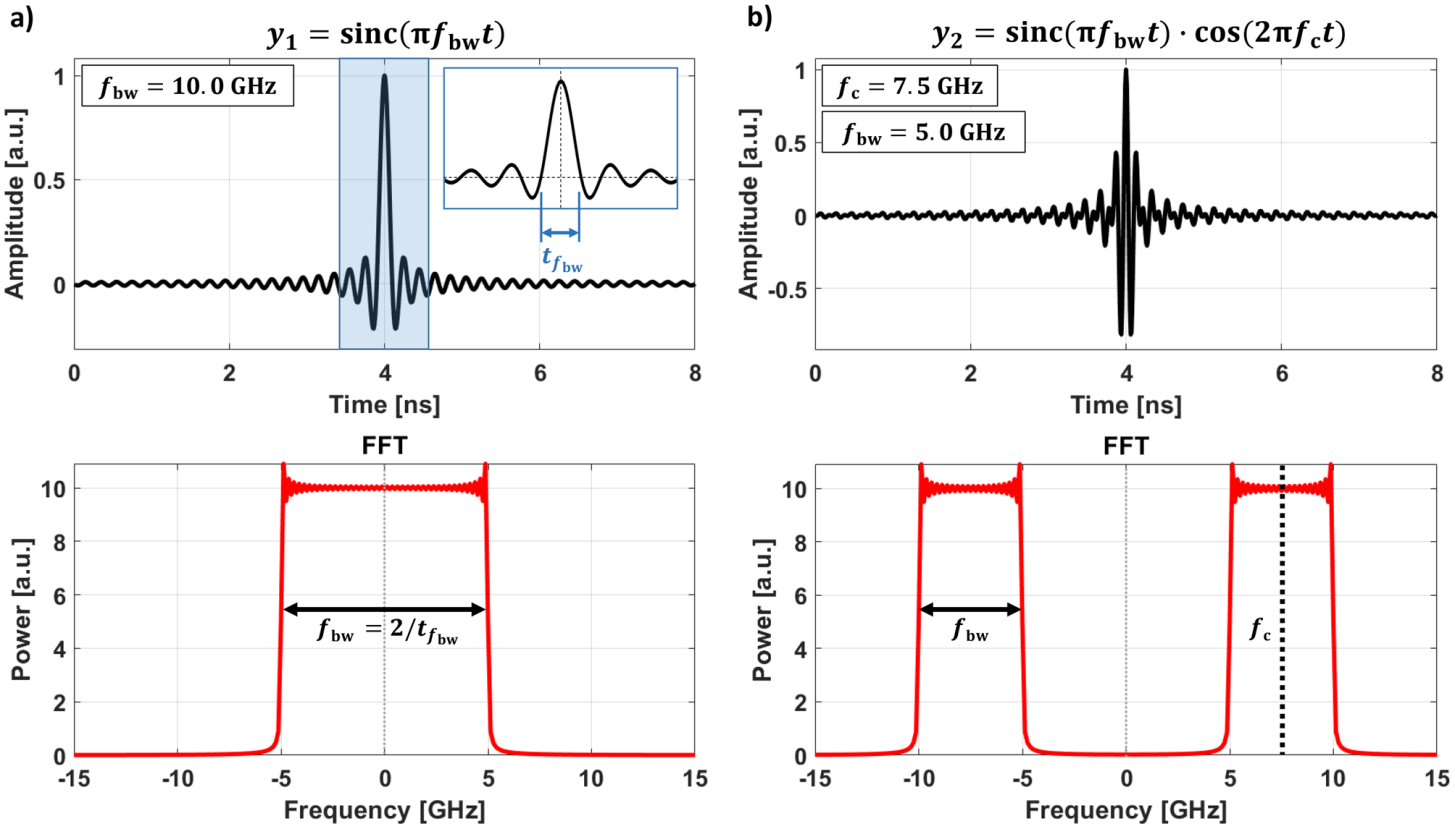
(**a**) Illustration of a usual Sinc function $y_{1}$ in time and frequency domain. The FFT in time reveals the frequency spectrum showing the characteristic rectangular shaped frequency band. $t_{f_{\mathrm {bw}}}$ defines the frequency bandwidth $f_{\mathrm {bw}}$. (**b**) Modified Sinc function $y_{2}$. A multiplication of a Sinc function with a cosine signal creates an adjustable frequency band as visible in the frequency spectrum below. The frequencies $f_{\mathrm {c}}$ and $f_{\mathrm {bw}}$ define the center frequency and the bandwidth, respectively.

In this work, we present an approach for measuring two dimensional spin wave dispersion relations *f*(*k*) within one single measurement including phase and amplitude maps in real space by time resolved STXM. Figure 1 schematically shows the used sample geometry. Spin waves are excited in the linear regime via a conventional coplanar waveguide [CPW, $\hbox {Ti}(3\hbox { nm})/\hbox {Cu}(120\hbox { nm}$)] on top of a $100\hbox { nm}$ thin yttrium-iron-garnet (YIG) film, which had been purchased from the company Matesy GmbH in Jena, Germany. The CPW were fabricated using lift-off processing after electron-beam lithography. The signal and ground lines of the CPW are $2.1\,\upmu \hbox {m}$ wide with a distance of $1.4\,\upmu \hbox {m}$ in between. A FFT of the CPW field distribution (c.f. supplementary material, Fig. S1) shows the excitation efficiency of all *k*-vectors. The blue arrows in Fig. 1 represent the induced oscillating radio frequency (RF) field. An external field $B_{\mathrm {ext}}$ is applied perpendicular and parallel to the *k*-vector defining the Damon–Eshbach (DE) and backward volume (BV) geometry, respectively. The resulting spin waves are illustrated in the middle of Fig. 1 for a selected area.

Previously, the common method of measuring *f*(*k*) using STXM was realized by continuous wave (CW) excitation and a sweep of RF frequencies while a static external field is applied^6,7,17^. Here, multiple measurements are required to reconstruct the dispersion relation. In contrast to CW excitation, we generate a modified Sinc function in time which defines a variable rectangular shape in the frequency domain (Fig. 2). Consequently, an arbitrary frequency range can be excited simultaneously with a uniform power distribution, revealing the full dispersion relation. A subsequent evaluation process for time resolved STXM movies relies on fast Fourier transformation (FFT) algorithms. In principle, a STXM movie represents a stack of magnetization images at different time steps. A time dependent FFT with a rectangular window function through each pixel of that image stack transforms this data into the frequency domain. Thus, each image represents now a specific frequency response with relative phase and amplitude information. As an additional step, the phase can be transformed into *k*-space by a spatial FFT to reveal the excited *k*-vectors. With this information for each frequency image, one can determine the full dispersion relation *f*(*k*). Further details about the evaluation process can be found elsewhere^17^. This method presents a single measurement approach to gain *f*(*k*) with *k*-sensitivity, phase and amplitude information in real space which drastically exceeds previously established experimental techniques.

Figure 2a shows a time domain signal of a conventional Sinc function

1 $$\begin{aligned} y_{1}=\frac{{\mathrm {sin}}(\pi f_{\mathrm {bw}}t)}{\pi f_{\mathrm {bw}}t}={\mathrm {sinc}}(\pi f_{\mathrm {bw}}t), \end{aligned}$$

where the frequency $f_{\mathrm {bw}}$ of the Sinc function defines the bandwidth $f_{\mathrm {bw}} = 10\hbox { GHz}$. A FFT in time is depicted below, pointing out the rectangular shaped frequency band.

2 $$\begin{aligned} f_{\mathrm {bw}}=\frac{2}{t_{f_{\mathrm {bw}}}} \end{aligned}$$

describes the frequency bandwidth $f_{\mathrm {bw}}$, where $t_{f_{\mathrm {bw}}}$ is the width in the time domain. The inset shows the definition of $t_{f_{\mathrm {bw}}}$.

A usual Sinc signal is centered at $0\hbox { GHz}$ and doesn’t exhibit a band-pass like frequency response with a higher and lower cut-off frequency, excluding undesirable low-frequency components. Figure 2b shows another approach from signal theory which permits a variable selection of different frequency ranges^18^:

3 $$\begin{aligned} y_{2}={\mathrm {sinc}}(\pi f_{\mathrm {bw}}t)\cdot {\mathrm {cos}}(2\pi f_{\mathrm {c}}t) . \end{aligned}$$

It introduces the multiplication of the Sinc signal with a cosine function, where $f_{\mathrm {c}}$ and $f_{\mathrm {bw}}$ define the center frequency and the bandwidth, respectively. In the shown case $f_{\mathrm {c}} = 7.5\hbox { GHz}$ and $f_{\mathrm {bw}} = 5\hbox { GHz}$. The frequency spectrum below indicates the effect of the multiplication.

**Figure 3:**
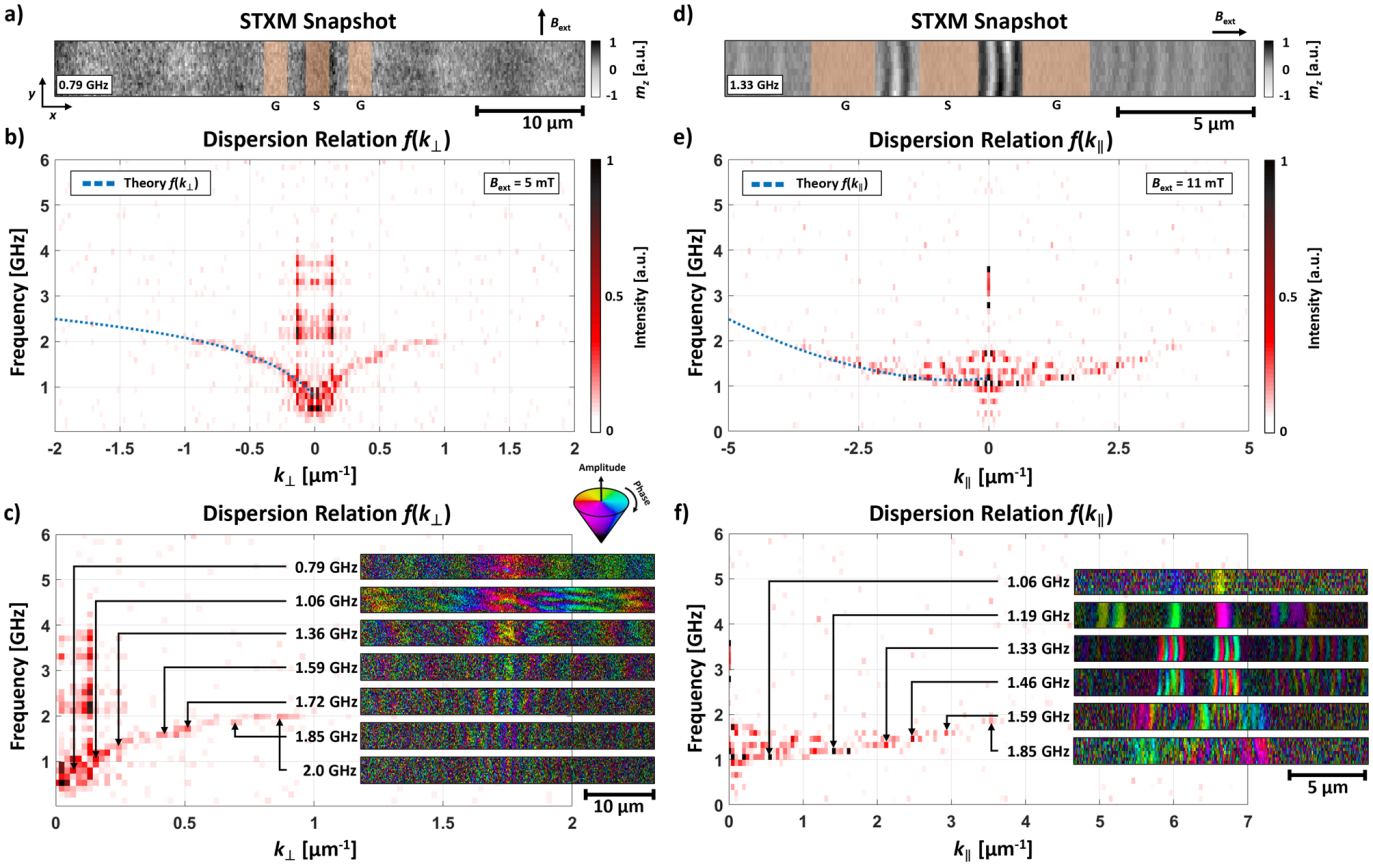
(**a**) Snapshot of a time resolved STXM movie revealing the $m_{z}$ component in DE geometry. The orange overlay illustrates ground (G) and signal (S) lines. (**b**) Experimental dispersion relation $f( k_{\perp } )$. The emerging DE mode branch (darked color) perfectly fits the theoretical prediction (blue dotted line). The constant *k*-vectors for frequencies above $f>2\hbox { GHz}$ are caused by electrical interference between the CPW and the photon detector. (**c**) Amplitude and phase maps of seven frequencies. The amplitude is encoded into brightness and the color represents the relative phase. As predicted, increasing the excitation frequency causes a decrease of spin wave wavelengths. (**d**) Snapshot of a time resolved STXM movie revealing the $m_{z}$ component in BV geometry. (**e**) Experimental dispersion relation $f( k_{\parallel } )$. The theoretical prediction (blue dotted line) fits the experimental results. Spin waves are excited between $f=1$–2 GHz with *k*-vectors up to $k_{\parallel } = 3.5\,\upmu \hbox {m}^{-1}$. (**f**) Amplitude and phase maps of six frequencies. Excited spin waves can primarily be observed between the signal and ground lines and next to the ground lines which is caused by the excitation and field geometry.

Measurements for experimental verification were carried out at the MAXYMUS endstation (UE46-PGM2 beamline) at the Helmholtz-Zentrum Berlin during multi-bunch operation mode. Magnetic contrast were achieved by circularly polarized X-rays and X-ray magnetic circular dichroism (XMCD). All measurements were performed at the $L_3$ absorption edge of iron at a photon energy of 707.8 eV to achieve maximum magnetic contrast. A Fresnel zone plate was used to focus the X-ray light down to a 20 nm spot and the sample was scanned through the focal spot to form an image. An avalanche photo diode that recovers between subsequent X-ray flashes (less than 1.5 ns) captures the transmitted intensity behind the sample. The temporal resolution is realized by a classical pump-probe setup where the synchrotron is used as a stroboscopic light source (probe). In doing so, only the FWHM length of the individual X-ray flashes determines the temporal resolution of the experiment. The multi-bunch operation mode provides a temporal resolution of approximately 50 ps. To determine the relative phase of the RF excitation (pump) a custom field-programmable gate array (FPGA) hardware is used. Each detected photon is sorted into a movie buffer accordingly. Additional information about the experimental setup and the timing scheme can be found elsewhere^19^.

The signal generation setup for time resolved STXM measurements relies on a Keysight M8195A arbitrary waveform generator (AWG), which is capable of creating software defined time domain signals up to 30 GHz and allows for the generation of complex waveforms as described in equation (3). Thereby, $y_{2}$ can be applied to excite magnons within a defined frequency band. For measurements in DE geometry, a scan size of $(45\cdot 2)\upmu \hbox {m}^{2}$ with a pixel size of $100\hbox { nm}$ in both *x*- and *y*-direction was used. BV scans were performed at a measuring window size of $(20\cdot 1)\upmu \hbox {m}^{2}$ with a pixel size of 67 nm in *x*-direction and 125 nm in *y*-direction. For a sufficient signal to noise ratio during these measurements, approximately 5 ms per pixel per time frame were used, leading to a total acquisition time of less than 40 min per movie. A sampling frequency of 20 GHz and $N=151$ time channels resulting in a reasonable resolution in the frequency domain for evaluation of the dispersion relation.

It is noteworthy that the sources of measurement uncertainties in our approach differ significantly from conventional techniques like BLS. Due to the lock-in detection with respect to the synchrotron, uncertainties and jitter in the electrical excitation do not lead to an error in frequency, but a reduced amplitude. Hence, the accuracy in frequency is better than 1‰  and a frequency resolution down to 25 kHz can be achieved. On the other hand, the resolution in wavevector *k* is only limited by the choice of imaging area and step size. Because the precision of the interferometrically controlled piezo stages is better than 2 nm, this source of uncertainty in *k* can be neglected.

With this experimental setup the dispersion relation *f*(*k*) of YIG is measured in DE and BV geometry. For the DE case, $f_{\mathrm {bw}} = 5\hbox { GHz}$ and $f_{\mathrm {c}} = 3\hbox { GHz}$ while an external field of $B_{\mathrm {ext}} = 5\hbox { mT}$ was applied in *y*-direction, i.e. $B_{\mathrm {ext}}\perp k$. The result of the modified Sinc excitation and the corresponding response of the magnonic system is shown in Fig. 3a–c. Here, $k_{\perp }$ ($k_{\parallel }$) denote the wavevector perpendicular (parallel) to $B_{\mathrm {ext}}$. A snapshot of a STXM movie is displayed in Fig. 3a in which ground (G) and signal (S) lines of the CPW are illustrated by an orange overlay. The greyscale represents the $m_{z}$ component of the magnetization. The dispersion relation $f( k_{\perp })$ results from a spatial FFT of each frequency slice into *k*-space and reveals spin wave excitation between $f=0.5$–2.0 GHz. Figure 3b depicts the resulting dispersion relation $f( k_{\perp })$. The emerging mode branch up to $k_{\perp }=1\, \upmu \hbox {m}^{-1}$ (darked color) perfectly fits the analytical predictions for thin films (saturation magnetization $M_{\mathrm {S}}=120\hbox { kA m}^{-1}$, exchange constant $\lambda _{\mathrm {ex}}=3.5\hbox { pJ m}^{-1}$, and gyromagnetic ration $\gamma = 176\hbox { rad ns}^{-1}\hbox { T}^{-1}$) in DE geometry indicated by the blue dotted line^20,21^. The peaks at constant wavevector $|k_{\perp }|=0.13\,\upmu \hbox {m}^{-1}$ appearing for several higher frequencies above $f=2\hbox { GHz}$ are a measurement artifact which is caused by electrical interference between the CPW and the photon detector. Visible *k*-vector excitation below $f=0.6\hbox { GHz}$ is also related to this effect. However, an inadvertently deposited magnetic particle on top of the signal line causes additional resonances leading to interference effects as depicted in Fig. 3c for $f=1.06\hbox { GHz}$.

Figure 3d–f shows the results of the Sinc excitation for BV geometry with $f_{\mathrm {bw}} = 4\hbox { GHz}$ and $f_{\mathrm {c}} = 0\hbox { GHz}$. The chosen center frequency $f_{\mathrm {c}}$ results in a conventional Sinc function without a band-pass like excitation. An external field of $B_{\mathrm {ext}} = 11\hbox { mT}$ was applied in *x*-direction. While Fig. 3d demonstrates a snapshot of $m_{z}$ at $f=1.33\hbox { GHz}$, Fig. 3e illustrates the dispersion relation $f( k_{\parallel })$. Spin wave excitation between $f=1$–2 GHz and up to $k_{\parallel }=3.8\,\upmu \hbox {m}^{-1}$ becomes visible and perfectly fits the indicated theory (blue dotted line). *k*-vectors above this line are related to standing spin waves below the signal and ground lines.

**Figure 4:**
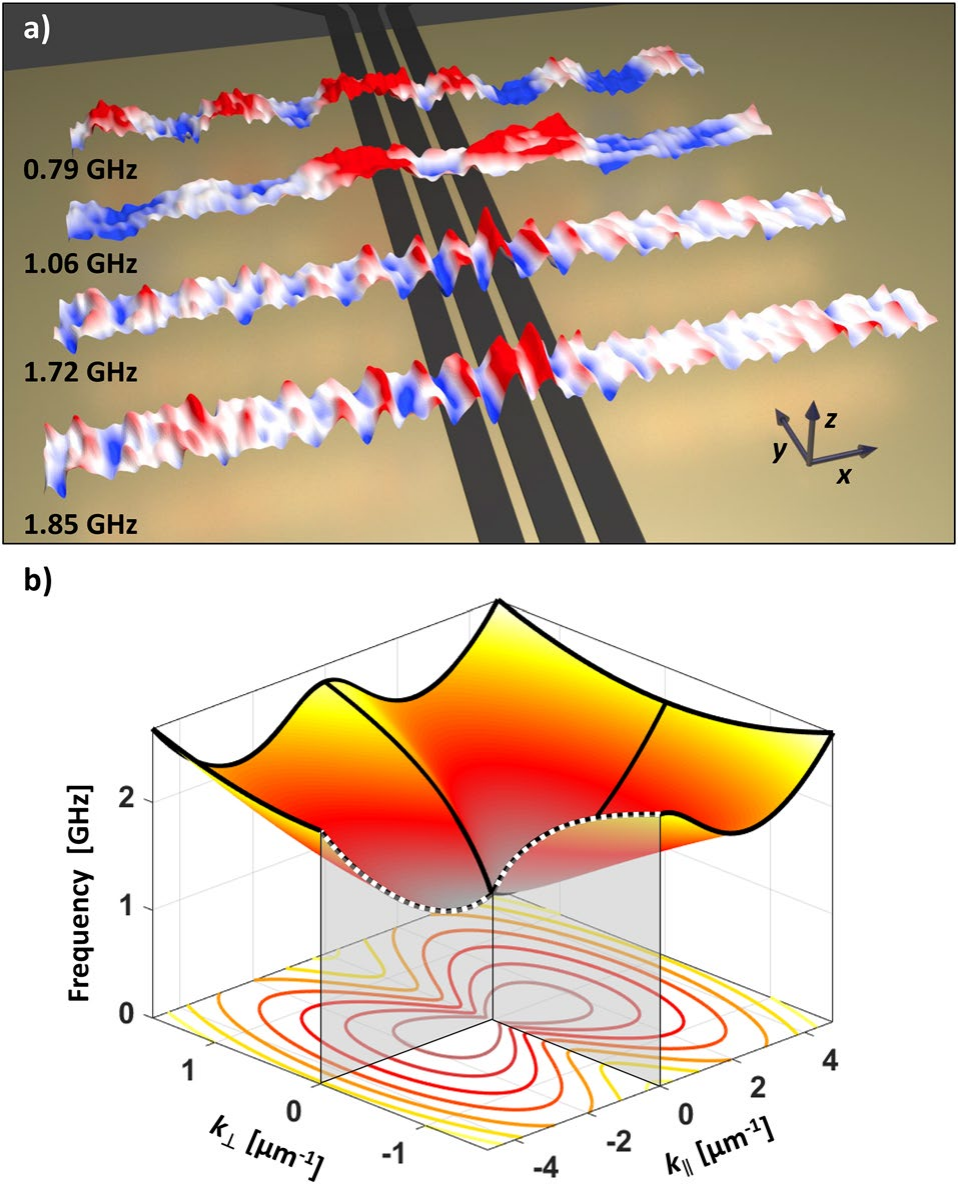
(**a**) Three dimensional illustration of spin waves excited by the modified Sinc approach. Magnons excited by frequencies between $f = 0.79$ and $1.85\hbox { GHz}$ are shown next to each other. (**b**) Three dimensional dispersion relation $f(k_{\perp , \parallel })$ of the YIG sample for $B_{\mathrm {ext}}=11\hbox { mT}$. The white dashed lines indicate the DE and BV mode branches presented in Fig. 3. Additionally, isofrequency curves of the dispersion relation are shown.

In Fig. 3f, six frequencies with their respective amplitude and phase information are depicted for the BV case. The amplitudes of spin waves in the area of the signal and ground lines of the CPW are reduced which is related to the excitation geometry and field distribution of the CPW. BV spin wave modes are excited by the coupling of the out-of-plane component of the induced alternating Oersted field with the out-of-plane component of the dynamic magnetization^22^. Thus, spin waves are excited mainly in the gaps and at the edges of the conductors. Furthermore, as visible at $f=1.33\hbox { GHz}$ multiple periods propagate between the signal and ground lines forming a standing spin wave pattern. This is caused by a slight mismatch of the impedance which leads to spin wave emission from the ground lines as it can be seen at $f=1.33\hbox { GHz}$. Additionally, each phase and amplitude map shows antiphase propagation which is caused by the BV geometry and the oscillating field distribution of the CPW. It is noteworthy that the dispersion relations $f(k_{\perp , \parallel })$ in Fig. 3 are evaluated for each geometry from one single time resolved STXM measurement within an acquisition time of 40 min.

One of the most powerful advantages of time resolved STXM is demonstrated in Fig. 3c,f. Due to the high temporal and spatial resolution, STXM allows for the analysis of different magnonic responses in real space as a function of multiple frequencies. Thus, every single frequency of the measured dispersion relation carries spatial amplitude and phase information. For the DE case in Fig. 3c, seven frequencies are presented with amplitude and phase. The amplitude is encoded in brightness, the color represents relative phase. The arrows indicate *k*-values at each frequency. The wavelength decreases with increasing excitation frequency. Figure 4a illustrates four frequencies between $f=0.79$ and $1.85\hbox { GHz}$, demonstrating the measured decreasing spin wave wavelength with increasing frequency in DE geometry. The lowest frequency reveals two spin wave periods while increasing frequency causes smaller wavelengths and amplitudes. The presented spin wave at $f=1.06\hbox { GHz}$ seems to possess a longer wavelength which however is caused by interference with the mentioned inadvertent magnetic particle. Furthermore, the non-reciprocity of the excited spin wave is well-visible^23,24^. For example, spin wave propagation at $f=1.85\hbox { GHz}$ is more pronounced in negative *x*-direction and diminished towards positive values.

We emphasize the versatility of probing the full dispersion relation with a Sinc excitation which is applicable for every magnonic structure that can be measured by time resolved STXM. Here, spin wave propagation emerges along one coordinate axis. The combination of both discussed measurements in DE and BV geometry reveals the anisotropic dispersion relation $f(k_{\perp , \parallel })$ as schematically illustrated in Fig. 4b for $B_{\mathrm {ext}}=11\hbox { mT}$. The theoretical dispersion relations $f(k_{\perp , \parallel })$ are depicted with dashed white lines indicating the DE and BV mode branch presented in Fig. 3. Even for single measurements of $k_{\perp , \parallel } > 0$ this method constitutes an efficient way to directly observe isotropic or anisotropic dispersion relations of magnonic ultra-thin films or complex structures beyond the discussed sample. Furthermore, isofrequency curves as indicated in Fig. 4b can be directly measured which has not been achieved so far. In principle, time resolved STXM with modified Sinc excitations can detect and directly image $f(k_{\perp , \parallel })$, including all excited magnons, in all directions, and hence, reveal full isofrequency curves. At the same time spin precession amplitudes and relative phases are extracted and non-reciprocity is evaluated.

In conclusion, we have demonstrated an approach for directly measuring the spin wave dispersion relation $f(k_{\perp , \parallel })$. For this purpose, an ultra-thin YIG film was investigated by time resolved STXM to validate the Sinc excitation and its impact on the magnonic response. Using modified Sinc functions consisting of a usual Sinc and a cosine signal allows for the generation of time signals with variable rectangular frequency bands. Therefore, a chosen range of frequencies can be simultaneously excited with uniform intensity. Additionally, phase and amplitude maps for each measured frequency are directly accessible. Thus, the two presented measurements for DE and BV geometry were performed in each case within one measurement at an acquisition time below 40 min exceeding the capabilities of other methods. The resulting dispersion relation in Fig. 3 perfectly fits the theoretical prediction. By combining signal theory and X-ray microscopy the investigation of spin wave excitation and propagation properties reveals outstanding possibilities for future magnonic ultra-thin structures.

## Supplementary information

Supplementary Figure S1.
