# Supplementary material for: Single shot acquisition of spatially resolved spin wave dispersion relations using X-ray microscopy
Source: Sci Rep. 2020 Oct 23;10:18146. doi: 10.1038/s41598-020-74785-4 (PMC7584636; doi:10.1038/s41598-020-74785-4)
Supplement: Supplementary file 1 — Supplementary Figure S1. [file 41598_2020_74785_MOESM1_ESM.docx]

Supplemental Material

**Single shot acquisition of spatially resolved spin wave dispersion relations using x-ray microscopy**

Nick Träger^1^, Felix Groß^1^, Johannes Förster^1^, Korbinian Baumgaertl^2^, Hermann Stoll^1, 3^,

Markus Weigand^1, 4^, Gisela Schütz^1^, Dirk Grundler^2^, and Joachim Gräfe^1, †^

^1^ Max Planck Institute for Intelligent Systems, 70569 Stuttgart, Germany

^2^ Laboratory of Nanoscale Magnetic Materials and Magnonics,

Institute of Materials, EPFL, 1015 Lausanne, Switzerland

^3^ Institute of Physics, Johannes Gutenberg University of Mainz, 55099 Mainz, Germany

^4^ Helmholtz-Zentrum Berlin für Materialien und Energie GmbH, 12489 Berlin, Germany

^Ɨ^ [graefe@is.mpg.de](mailto:graefe@is.mpg.de)

| 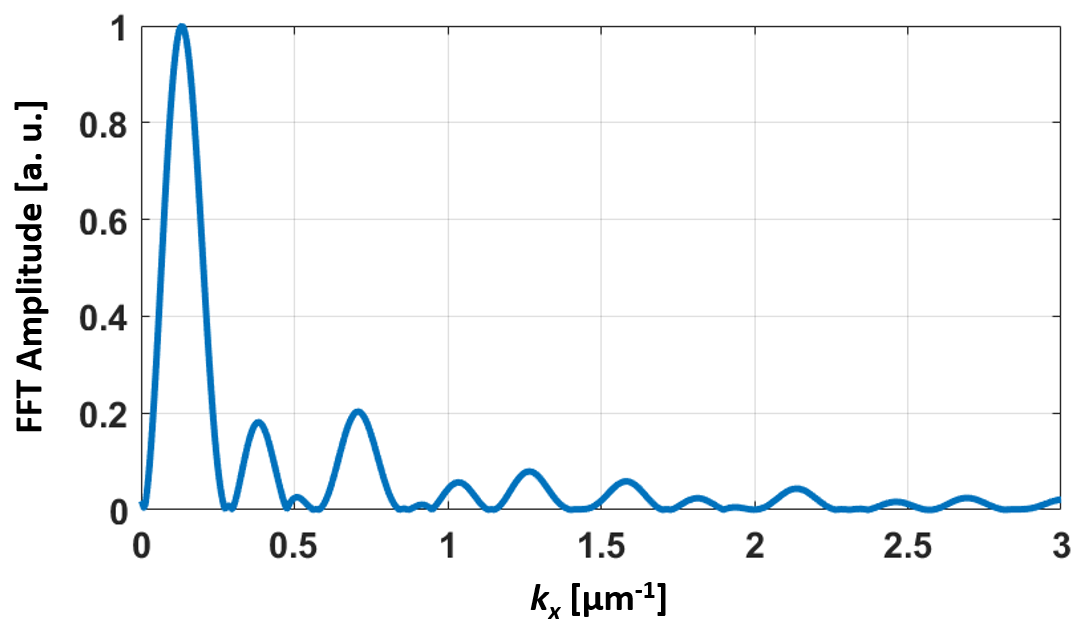 |
| --- |
| ***Supplementary Figure S1****: Excitation efficiency of the CPW. The FFT of the spatial profile (x-component of the exciting Oersted field) reveals all available k-vectors.* |
